# Antibiogram studies of *Escherichia coli* and *Salmonella* species isolated from diarrheal patients attending Malam Mande General Hospital Dutsin-Ma, Katsina State, Nigeria

**DOI:** 10.11604/pamj.2020.37.110.24851

**Published:** 2020-10-01

**Authors:** Ayodele Timilehin Adesoji, Ahmad Mansur Liadi

**Affiliations:** 1 Department of Microbiology, Faculty of Life Sciences, Federal University Dutsin-Ma, Katsina State, Nigeria

**Keywords:** *Escherichia coli*, *Salmonella spp*, multidrug resistant, antibiogram, diarrhea, stool samples

## Abstract

**Introduction:**

antibiotics resistant bacteria (ARB) is a worldwide problem. Information on ARB associated with diarrheal stool samples from Dutsin-Ma, Katsina State, Nigeria is scare.

**Methods:**

this study examines 41 stool samples of diarrhea patients from a selected hospital in Dutsin-Ma. Questionnaires were used to collect demographic information and used antibiotics. Bacteria isolation and antibiotics susceptibility tests were determined using standard microbiological techniques. Multidrug resistant (MDR) bacteria were selected based on resistant to ≥3 classes of antibiotics.

**Results:**

twenty bacteria that include Escherichia coli (n = 15) and Salmonella spp. (n = 5) were isolated. Pediatric age group (0-5 years) showed highest prevalence of 73.3 and 60% respectively. Illiterate patients showed highest (60%) frequency of Salmonella spp. Tetracycline was mostly observed for treating diarrhea among patients; high resistance to amoxicillin (80%), ampicillin (100%) and tetracycline (73.3%) was noticed in E. coli. To each of amoxicillin and ampicillin, 100% resistance was observed among Salmonella spp. Two and one MDR E. coli and Salmonella spp. were identified respectively.

**Conclusion:**

high occurrence of studied bacteria among infants and aged adults coupled with some displaying MDR characteristics calls for urgent public health attention, hence, comprehensive studies are needed for the determination of molecular epidemiology of these bacteria for public health surveillance.

## Introduction

Diarrhea is defined as a disease condition characterized with the passage of three or more loose stools per day or more frequent passage than is normal for a healthy individual [1]. It symptoms are characterized with gastroenteritis, stomach cramps, vomiting, and dehydration caused by a host of bacterial, viral and parasitic organisms [1]. WHO reported increased episode of diarrhea every year in developing countries (including Nigeria) [1]. As at the end of 2015, Nigeria still ranked second among the top 15 countries with high child mortality due to diarrhea and pneumonia [2]. Diarrheal disease is a leading cause of mortality and morbidity across the globe [3]. Diarrheal disease affects all age groups, infants and children below the age of five and these age groups are more predisposed to diarrheal diseases than any other age groups [3]. Infective bacterial diarrhea is caused by variety of organisms which includes *Escherichia coli, Campylobacter spp., Clostridium perfringens, Salmonella spp., Shigella spp*., among others [4].

*Escherichia coli* is a Gram-negative, rod-shaped, facultative anaerobic bacterium that belong to the family Enterobacteriaceae [5]. *E. coli* strains implicated in diarrhea are referred to as diarrheagenic *E. coli* (DEC). DEC are one of the most important of the various etiological agents of diarrhea, which includes enterotoxigenic *E. coli* (ETEC), enteropathogenic *E. coli* (EPEC), enteroaggregative *E. coli* (EAEC), enteroinvasive *E. coli* (EIEC), enterohemorrhagic *E. coli* (EHEC), as well as, diffusely adherent *E. coli* (DAEC) among others. These pathotypes differ regarding their preferential host colonization sites, virulence mechanisms, and the ensuing clinical symptoms and consequences, and are identified using molecular techniques including primers that is specific to each pathotypes [6]. *Salmonella*, a genus of Gram-negative rod shaped bacteria of the family Enterobacteriaceae, causes a wide range of human diseases, such as enteric fever, gastroenteritis, endocarditis, and bacteraemia. It constitutes a major public health burden and represents a significant health cost in many countries. *Salmonella* serovars such as *Salmonella* serovar Typhimurium, *Salmonella* serovar Paratyphi, and *Salmonella* serovar Choleraesuis among others are grouped as Non-typhoidal *Salmonella* (NTS) which are also known to be implicated in the cause of diarrhea [7].

Antibiotic resistance pattern exhibited by diarrheagenic *Escherichia coli* strains and *Salmonella spp*. are quite uniquely associated with the presence of some specific antibiotic resistance genes such genes encode resistance to tetracycline (*tetA*and *tetB*), ampicillin (CITM), and chloramphenicol (*cat1* and *cmlA*) among others. Antibiotic resistant diarrheagenic *E. coli* and *Salmonella* species cause severe diarrheal disease, and could be associated with treatment failures among diarrheal patients resulting in increasing mortality associated with infective bacterial diarrhea [8]. Diarrheal diseases remain among the most common causes of mortality and morbidity particularly in developing countries (including Nigeria) [3]. Risk factors related to inadequate water, sanitation, poor personal hygiene, and malnutrition remain unacceptably high [9]. Consumption of contaminated food and water with pathogenic (*Escherichia coli* and *Salmonella spp*.) can lead to infective bacterial diarrhea [10]. Antibiotic resistance is a major global public health concern, particularly in settings where few treatment options are available in the treatment of infective bacterial diarrhea [11]. However, there is paucity of information on antibiogram studies of *Escherichia coli* and *Salmonella spp*. associated with diarrheic stools of patients in Dutsin-Ma, Katsina State, Nigeria, coupled with the fact that antibiotic susceptibility profiles of bacterial isolates of diarrheal patients is not provisional during the period of sampling at the selected hospital, which makes it imperative to carry out this study. This research is, therefore, aimed at determination of antibiogram of *Escherichia coli* and *Salmonella spp*. associated with stool samples of diarrhea patients attending Malam Mande General Hospital, Dutsin-Ma, Katsina State, Nigeria.

## Methods

**Description of study area:** this study was conducted in Dutsin-Ma, Dutsin-Ma is a Local Government Area (LGA) in Katsina State, Nigeria. It is located on latitude and longitude 12°27'18''N, 7°29'29''E respectively. The LGA has an area of 527 km^2^and population of 169,671 as of 2006 census, with Zobe Dam lying to the south of the town [12]. The inhabitants of the local government are predominantly Hausa and Fulani by tribe. Their main occupation is farming and animal rearing.

**Ethical approval:** ethical approval was obtained from Katsina State ministry of health for permission to obtain diarrhea samples from patients at the selected hospital with approval number: MOH/ADM/SUB/1152/1/258. Informed consent was obtained from patients, as well as parents or guardians of the children that were sampled.

**Sample collection:** forty one diarrheic stool samples were collected between July and September, 2019 in universal specimen collection tubes from diarrheal patients at Malam Mande General Hospital, Dutsin-Ma, and transported on ice packed to the laboratory of Department of microbiology, Federal University, Dutsin-Ma for microbiological analysis.

**Questionnaires:** structured questionnaires were administered to patients or care giver of children that participated in this study for collection of demographic information, antibiotics and medications used in the treatment of diarrhea, with patients categorical classification (in patients or out patients) that might influence laboratory results obtained.

### Isolation of bacteria

**Isolation of *Escherichia coli*:** sterile swab stick was used in streaking diarrheic stool samples collected from diarrheal patients on sterile Eosin Methylene Blue Agar and MacConkey Agar for isolation of *Escherichia coli* [4, 13], subsequently the plates was incubated at 37°C for 24 hours in an incubator. Afterwards, the plate was observed for colony formation after 24-48 hours of incubation. In other to obtain a distinct colony, bacteria was subcultured on EMB and MacConkey agar. Distinct green metallic sheen colonies and pink colonies was aseptically picked, streaked and stored on Nutrient agar slant for further biochemical characterization. Colonial appearance such as size, shape, color, elevation, and differential characteristics such as pigmentation, lactose fermentation on MacConkey agar and Gram staining were carried out to further identify the isolates.

**Isolation of *Salmonella* species:** one (1) gram of diarrheic stool sample from diarrheal patients was enriched in 5ml of Rappaport Vassiliadis R10 broth at 37°C for 24 hours [14] followed by subculturing on both MacConkey Agar and *Salmonella-Shigella*(SS) agar and the plates was incubated at 37°C for 24 hours [4]. Afterwards, the plates were observed for colony formation after 24-48 hours of incubation. Pure cultures were prepared from *Salmonella* like colonies, i.e. colorless colonies on MacConkey Agar, and colorless colonies with black spot at the center on SS Agar, was aseptically picked and stored on Nutrient agar slant for further biochemical characterization.

**Bacteria characterization and identification:** *Escherichia coli* and *Salmonella* species isolated were subjected to various biochemical tests such as: gram-stain, motility test, urease test, indole test, methyl-red, vogues proskauer test, citrate test, oxidative fermentation test, triple sugar iron agar test for biochemical characterization, and identified according to the method of Cowan and Steel [15].

**Antibiogram studies:** *Escherichia coli*. and *Salmonella* species isolates were subjected to antimicrobial susceptibility testing using disk diffusion method. A bacterial lawn was prepared by transferring overnight grown bacteria colonies to a glass tube containing 5 ml sterile normal saline water with a sterile inoculating loop. The suspension was vortexed and visually matched with 0.5 MacFarland standard for turbidity. Sterile cotton tipped swab was immersed in the suspension, excess fluid was removed by rolling the swab on the upper part of the tube, and spread onto Mueller Hinton agar (Oxoid, UK) to obtain a semi-confluent growth. Disks impregnated with predetermined amounts of antibiotics was dispensed onto the bacterial lawn and the plates was incubated for 18-24 hours at 37°C. After the incubation, the diameter of the inhibition zones was measured in millimeters using ruler and interpreted as sensitive, intermediate, and resistant using the criteria described by the Clinical and Laboratory Standards Institute [16]. The antibiotics disc (Oxoid, UK), that was used include: amoxicillin (10μg), ampicillin (10μg), ceftriaxone (30μg), chloramphenicol (30μg), ciprofloxacin (10μg), nalidixic acid (30μg), imipenem (10μg), streptomycin (10μg), and tetracycline (30μg). These antibiotics are used in humans for treatment of gram negative pathogens.

## Results

In this study, a total of 20 (48.8%) bacteria isolates comprising of 15 (36.6%) *Escherichia coli* and 5 (12.2%) *Salmonella spp*. were isolated among 41 (100%) diarrheic stools samples (Table 1). The percentage occurrence of bacteria isolate was higher in female 11 (55%) than their male 9 (45%) counterpart. It was also observed that 21 (51.2%) stools samples showed no bacterial growth (Table 1). In Table 2, *Escherichia coli* 11 (73.3%) and *Salmonella spp*. 3 (60%) were isolated respectively in patients between 0-5 years old (pediatric group). We also isolated 6.7% and 40% of these bacteria respectively in patients of aged ≥60 (elderly). However, among patients aged ≤ 19 (young adult) only *E. coli* (20%) was isolated. It was also observed that female patients showed highest occurrence of *E. coli* (8/15) and *Salmonella spp*. (3/5). Table 3shows male patients as the highest (58.5%) with diarrhea symptom compared to their female counterpart (41.5%) in this study. To limit bias no patient was excluded except a few patients who refused to participate. A total of 29 (70.7%) outpatients participated in the study while 12 (29.3%) were in patients. The frequency of isolation for *Salmonella spp*. was higher (60%) among in-patient than outpatients (40%). The frequency of isolation for *Salmonella spp*. was also higher (60%) among illiterate than any other group in the study population. However, from the administered questionnaire, antibiotics 21 (51.2%), remain the sole treatment option considered in treatment of diarrhea among the study population, followed by zinc tablet supplement 13 (31.7%) and least considered treatment option is the anti-diarrheal agent 7 (17.1%).

**Table 1:** percentage distribution of bacterial isolates among the study population

Bacteria	No of Samples Positive	(%)	No of Samples Negative	(%)	Total Sampled	(%)	Sex				Total	(%)
							Male Positive	(%)	Female Positive	(%)		
***Escherichia coli***	15	(36.6%)	26	(63.4%)	41	(100%)	7	(46.7%)	8	(53.3%)	15	(100%)
***Salmonella spp*.**	5	(12.2%)	36	(87.8%)	41	(100%)	2	(40%)	3	(60%)	5	(100%)
**Total**	20	(48.8%)	21	(51.2%)	41	(100%)	9	(45%)	11	(55%)	20	(100%)

**Table 2:** age and gender distribution of *Escherichia coli* and *Salmonella spp*. isolates among the study population

	*Escherichia coli*			*Salmonella* spp.		
Age group (years)	Male	Female	Total (%)	Male	Female	Total (%)
**0-5 (Pediatric group)**	6	5	11 (73.3%)	2	1	3 (60%)
**≤19 (Young adult)**	1	2	3 (20%)	0	0	0 (0%)
**20-39 (Adult)**	0	0	0 (0%)	0	0	0 (0%)
**40-59 (Post adult)**	0	0	0 (0%)	0	0	0 (0%)
**≥60 (Elderly/Seniors)**	0	1	1 (6.7%)	0	2	2 (40%)
**Total**	**7**	**8**	**15 (100%)**	**2**	**3**	**5 (100%)**

**Table 3:** socio-demographic distribution of bacterial isolates of the study population

		Frequency Number	*Escherichia coli*	*Salmonella* spp
		N (%)	N (%)	N (%)
**Total**		**41 (100%)**	**15 (100%)**	**5 (100%)**
Gender	Male	24 (58.5)	7 (46.7)	2 (40)
	Female	17 (41.5)	8 (53.3)	3 (60)
Severity	In patient	12 (29.3)	5 (33.3)	3 (60)
	Out patient	29 (70.7)	10 (66.7)	2 (40)
Education Level	Illiterate	19 (46.3)	8 (53.3)	3 (60)
	Basic 1-6	8 (19.5)	3 (20)	1 (20)
	Jss1-Sss3	10 (24.4)	4 (26.7)	1 (20)
	Undergraduate	4 (9.8)	0 (0)	0 (0)
Medication	Zinc tablet and ORS	13 (31.7)	0 (0)	0 (0)
	Antibiotics	21 (51.2)	0 (0)	0 (0)
	Anti-diarrheal agent	7 (17.1)	0 (0)	0 (0)

All the fifteen (15) isolates of *Escherichia coli* were susceptible to chloramphenicol (100%) while highest frequency of resistance was observed to ampicillin 15 (100%) follow by amoxicillin 12 (80%), while percentage resistance to tetracycline and imipenem was observed to be 73.3% and 46.7% respectively (Table 4). Furthermore, all the 5 *Salmonella spp*. isolates were susceptible to each of chloramphenicol and ciprofloxacin (100%) while 80 and 60% were susceptible to each of nalidixic acid and streptomycin respectively. In addition, *Salmonella spp*. showed 5 (100%) resistance to each of ampicillin and amoxicillin, followed by 3 (60%) to tetracycline and 2 (40%) to imipenem. Multidrug Resistance (MDR) bacteria were selected based on resistance to either three classes or over three classes (≥3) of antibiotics. Two (2) *E. coli* isolates displayed MDR characteristics as they were resistance to more than 3 different classes of antibiotics which include aminoglycosides, cephalosporins, fluoroquinolones, penicillins, quinolones and tetracyclines. However, only one *Salmomella spp*. displayed MDR characteristics as they were resistant to 3 different classes of antibiotics which include aminoglycosides, penicillins, quinolones and tetracyclines. One isolate of both *E. coli* and *Salmonella spp*. was found resistant to 3 similar antibiotics which include tetracycline, nalidixic acid, and streptomycin (Table 5).

**Table 4:** antibiotic susceptibility pattern of *Escherichia coli* and *Salmonella spp*. isolates from diarrheic stools among the study population

	Number (%)	Number (%)	Number (%)
Antimicrobial Agents	Resistant (R)	Intermediate (I)	Sensitive (S)
	***Escherichia coli* (n=15)**		
Amoxicillin	12 (80)	1 (6.7)	2 (13.3)
Ampicillin	15 (100)	0 (0)	0 (0)
Ceftriaxone	5 (33.3)	4 (26.7)	6 (40)
Chloramphenicol	0 (0)	0 (0)	15 (100)
Ciprofloxacin	1 (6.7)	3 (20)	11 (73.3)
Imipenem	7 (46.7)	2 (13.3)	6 (40)
Nalidixic acid	1 (6.7)	3 (20)	11 (73.3)
Streptomycin	3 (20)	1 (6.7)	11 (73.3)
Tetracycline	11 (73.3)	3 (20)	1 (6.7)
	***Salmonella spp*. (n=5)**		
Amoxicillin	5 (100)	0 (0)	0 (0)
Ampicillin	5 (100)	0 (0)	0 (0)
Ceftriaxone	1 (20)	1 (20)	3 (60)
Chloramphenicol	0 (0)	0 (0)	5 (100)
Ciprofloxacin	0 (0)	0 (0)	5 (100)
Imipenem	2 (40)	3 (60)	0 (0)
Nalidixic acid	1 (20)	0 (0)	4 (80)
Streptomycin	1 (20)	1 (20)	3 (60)
Tetracycline	3 (60)	1 (20)	1 (20)

**Table 5:** multidrug resistant profiles of 3 bacteria isolates

Bacteria	No of MDR	Resistant Patterns
*Escherichia coli*	Two (2)	AML, AMP, CEFT, STREP, TET
		AML, AMP, CEFT, CIPX, NA
*Salmonella spp*.	One (1)	AML, AMP, NA, STREP, TET

KEYS; AML=Amoxicillin, AMP=Ampicillin, CEFT=Ceftriaxone, CIPX=Ciprofloxacin, NA=Nalidixic acid, STREP=Streptomycin, TET=Tetracycline, MDR=Multi Drug Resistant profile

Table 6, shows routinely, commonly used antibiotics among the study population which may have contributed to the vast increase in antibiotics resistant among the bacteria, emergence of MDR that may lead to treatment failure, antibiotic mediated diarrhea and false negative bacterial culture from patient sampled. Majority of the study population lack basic knowledge on the name and meaning of antibiotics. In addition tetracycline and metronidazole remain the most commonly used antibiotics in the treatment of diarrhea among the patient sampled in the study population.

**Table 6:** distribution of antibiotics taken by the sampled patients in the last 30 days before hospital visit

				Age	
Antibiotics used in the last 30 days		N	%	Mean	Range
Yes		17	41.5	21.30	0-60
No		24	58.5	10.04	0-65
	**Unreported**	24	58.5	10.04	0-65
**Antibiotics class**	**Generic name**				
Penicillins	Ampiclox	1	5.88	14.00	
	Ampicillin	2	11.76	18.5	14-23
Nitroimidazole	Metronidazole	6	35.29	30.90	6-60
Cephalosporins	Ceftriaxone	1	5.88	60.00	
Fluoroquinolones	Ciprofloxacin	2	11.76	18.00	2-34
Macrolides	Azithromycin	1	5.88	26.00	-
Tetracyclines	Tetracycline	7	41.17	18.10	2-40
Aminoglycosides	Gentamicin	1	5.88	22.0	-

## Discussion

In this study, 36.6% occurrence of *Escherichia coli* was observed among the study population (Table 1), which is lower compared to 41.4% occurrence of *E. coli* reported by Korie *et al*. [13], and 59% occurrence of *E. coli*from diarrheic stools reported by Dormanesh *et al*. [17] in a study conducted in Enugu, Nigeria and Iran respectively. Nan-Ok *et al*. [4] also reported 22.0% of *E. coli*from diarrheic stool specimen from a study conducted in Korea. However, 12.2% occurrence of *Salmonella spp*. was observed in this present study, which is higher than 1.2% and 5% occurrence of *Salmonella spp*. reported by Nair *et al*. [18] and Mzungu *et al*. [19] in a study conducted in India and Nigeria respectively. Tesfahun *et al*. [20] reported 10.8% occurrence of *Salmonella spp*. from diarrheic stools in a study conducted in Ethiopia while Kabir *et al*. [21] reported 17% occurrence of *Salmonella spp*. from stool specimen of patients with gastroenteritis in a study conducted in Lagos, Nigeria. This disparity may be attributable to differences in the study designs, patients´ selection, differing environmental condition and behavioral pattern in those regions.

Moreover, in this study the highest frequency of bacterial isolation from diarrheic stools where among the subjects in the age group 0 to 5 years (pediatric group) (Table 2) and this is similar to reports from Nigeria Korie *et al*. [13], Mzungu *et al*. [19], Abdullahi *et al*. [22], Sule *et al*. [23], Senegal Thiam *et al*. [9] and Saudi Arabia Al-jurayyan *et al*. [24]. This could be because diarrhea could result from hand contamination among these children especially while playing on the ground, playing with toys or other objects, and unknowingly putting their dirty finger in their mouth. In addition, the risk of ingesting contaminated materials among this age group is high, especially in unhygienic environments. The low frequency of isolation among young adult within the age group of ≤ 19 might be associated with the development of immunity or loss of receptor for specific adhesion molecules. Similarly, stools positive culture for bacteria observed among seniors within the age category of ≥ 60 might be associated with the fact that surveys on seniors have found that non negligible proportion of older adult do not follow recommended food safety practices, which makes them vulnerable to gastroenteritis associated with the studied bacteria i.e. *Salmonella spp*. There was higher frequency of stool culture positive for bacteria among male within 0 to 5 than their female counterpart. This is in contrast with study of Oliver *et al*. [25] from Kenya where diarrheic stool positive culture for bacteria was higher in female than their male counterpart within the same age category, this might be associated with differences in study design and geographical location. Diarrheic stools specimens from females within the age category ≤19 (Young adult) and ≥ 60 (Elderly) tested positive for bacterial culture than their male counterpart. This might be due to random selection of diarrhea patient that participated in this study.

Furthermore, this study indicated that *E. coli* isolates showed high resistance rate to ampicillin (Table 4). This findings is in agreement with reports from Thailand, Wilunda and Panza [26], Kenya, Sang *et al*. [27] and Nigeria, Korie *et al*. [13]. The antibiotics sensitivity pattern shows high sensitivity to ciprofloxacin and chloramphenicol which is similar to study conducted in Lagos, Nigeria by Alabi *et al*. [28], and Bahir Dar, Ethiopia, Ayrikim *et al*. [29] and Sang *et al*. [27], and Oman, Ali *et al*. [30]. In addition, high sensitivity of *Salmonella spp*. isolates to chloramphenicol and ciprofloxacin observed in this study is similar to reports by other authors from Katsina, Nigeria (Mzungu *et al*. [19]), Lagos, Nigeria (Kabir *et al*. [21]), Enugu, Nigeria (Ogbu *et al*. [31]) and Ethiopia (Beyene and Tasew [32]). These increase in resistance may be attributed to the widespread misuse of this drug, coupled with the fact that they are cheap, people can purchase these drugs from the open market in the study area without physician prescription. Increased and high susceptibility to chloramphenicol might be attributed to the cost of the antibiotics, it may be expensive compared to ampicillin, amoxicillin and tetracycline which cannot be easily afforded by majority of the people from the study area, and therefore this may have contributed to the effectiveness of the chemotherapeutic agent in treatment of diarrhea caused by *Salmonella spp*.

High resistance of bacteria (*E. coli* and *Salmonella spp*.) to beta lactam antibiotics observed in this study (Table 4) is also in agreement with high resistance of bacteria to beta lactam antibiotics reported by Adesoji and Ogunjobi [33] from non-clinical samples in a study conducted in Nigeria. In addition, occurrence of tetracycline and streptomycin resistance among MDR bacteria (*E. coli* and *Salmonella spp*.) observed in this study (Table 5) is similar to tetracycline resistant MDR and aminoglycoside resistant MDR bacteria reported by Adesoji *et al*. [34] and Adesoji *et al*. [35] respectively from environmental samples in a study conducted in southwestern Nigeria. This might be explained by the fact that indiscriminate use of antibiotics may have contributed to vast emergence and spread of multidrug resistant bacteria from both clinical and non-clinical environment.

From this present study, the frequency of isolation was highest with (3, 60%) *Salmonella* species isolates from hospitalized patients (Table 3), this is in contrast to (77, 5.4%) *Salmonella* species isolates from hospitalized patients reported by Thompson *et al*. [36], in a study conducted in Vietnam. Furthermore, in relation to educational status and frequency of isolation, this study indicated highest isolation (3, 60%) of *Salmonella spp*. was observed among illiterates (Table 3). This result is consistent with earlier studies conducted by Sofia *et al*. [37] and Tesfahun *et al*. [20]. Education is vital to create awareness in the community with regard to the mechanism of management of infectious diarrhea and control of other factors that lead to this disease. In this study, increased use of tetracycline (41.17%) indicated among the sampled patients (Table 6) might have contributed to high percentage of resistance to tetracycline (14, 70%) observed in this study. The result of high negative stool culture (Table 1) might be as a result of pretreatment with antibiotics among the sampled patients within the last 30 days. This is similar to report by Korie *et al*. [13]. In addition this might also be due to the fact that pathogens such as parasites and viruses are also implicated in diarrheal diseases.

Antidiarrheal agent contain Loperamide hydrochloride sold and known to the sampled patients under the brand name of (Lemotil and diarrhea stop) which is used in the treatment of antibiotics associated diarrhea, was found to be the least treatment options considered for treatment of diarrhea in the study area (Table 3). With the vast increase of resistance to commonly used antibiotics associated with the studied bacteria, antidiarrheal agent could be considered as an effective alternative medication to antibiotics with respect to diarrhea diseases. However, susceptibility of bacteria isolates to chloramphenicol and fluoroquinolones (ciprofloxacin) observed in this study (Table 4), coupled with its increased effectiveness in in-vitro antibiotic susceptibility profile reported by Beyene and Tasew [32] in Ethiopia, Kanyina *et al*. [38] in Kenya, and Mzungu *et al*. [19] in Nigeria, among others showed that these chemotherapeutic agents could be effective in treatment of *Escherichia coli* and *Salmonella* species associated diarrhea in those region.

## Conclusion

In conclusion, from this present study, *Escherichia coli* and *Salmonella spp*. were frequently isolated among pediatric age groups (0-5) with prevalence rate of 73.3% and 60% respectively. However, the results of antibiotic susceptibility tests in this study showed high level of resistance among these isolates especially to ampicillin (100%), amoxicillin (80%) and tetracycline (70%) making them completely unreliable in the management of *Escherichia coli* and *Salmonella spp*. associated diarrhea in the study area. It was also observed that 15% of bacteria displayed multidrug resistant characteristics. Hence, comprehensive studies are needed for the determination of the molecular epidemiology of these resistant bacteria for public health surveillance.

### What is known about this topic

- Escherichia coli and Salmonella species remains important aetiological agents associated with infective bacterial diarrhea;
- Emergence of multidrug resistant E. coli and Salmonella species implicated in infective bacterial diarrhea is of public health significance.

### What this study adds

- Identified that E. coli and Salmonella spp. are more predominant among pediatric age group;
- Identified that male patients exhibit highest symptoms of diarrhea compared to their female counterpart;
- Identified that MDR E. coli and Salmonella spp. that are of immerse public health significance can be isolated from this study.
